# Conduction System Pacing: Where Are We in 2025?

**DOI:** 10.19102/icrm.2025.16015

**Published:** 2025-01-15

**Authors:** Imran Niazi

**Affiliations:** 1 Aurora St. Lukes Medical Center, Advocate Health, Milwaukee, WI, USA; 2 University of Wisconsin School of Medicine and Public Health, Madison, WI, USA

**Keywords:** Cardiomyopathy, conduction system pacing, left bundle branch block

Conduction system pacing (CSP) for the prevention of right ventricular (RV) pacing–induced cardiomyopathy and for resynchronization after left bundle (LB) branch block (LBBB) remains an important focus of research. In particular, LB pacing appears to be the most clinically applicable therapy as it avoids the disadvantages of His-bundle pacing (sensing challenges, higher thresholds, distal conduction system disease) and offers a simpler and quicker implant procedure.

The terminology used to describe the various forms of LB pacing has become increasingly confusing, though some degree of agreement was reached after the recent European Heart Rhythm Association (EHRA) consensus statement on CSP.^1^ In brief, left ventricular (LV) septal pacing (LVSP) is considered distinct from the well-accepted selective and non-selective left bundle branch pacing (S-LBBP and NS-LBBP, respectively). The left-sided conduction system is directly engaged selectively (S-LBBP) or with the additional activation of the subendocardial myocardium (NS-LBBP), leading to more rapid activation of the LV compared to RV pacing in both cases. In patients with LBBB, delayed activation of the basal and posterior lateral walls is counteracted when there is no disease of the conduction system distal to the pacing site. Rapid LV activation is reflected in an abrupt shortening of the conduction time from the pacing stimulus to the R-wave peak in the lateral chest leads V5 and V6 (V6-RWPT, loosely referred to as LV activation time) by at least 10 m, when the LB is engaged. Additionally, V6 activation now precedes V1, and the V6–V1 interpeak interval is prolonged to >44 ms (S-LBBP) and 33 ms (NS-LBBB) instead of being almost simultaneous with septal pacing. Early LV activation produces a right bundle branch block (RBBB)-like pattern with qR, QR, or RsR in V1 with S-waves in V5 and V6. In LVSP, the LB is not directly engaged, and there is no abrupt shortening of Stim–V6, although an RBBB-like pattern is still seen on electrocardiogram (ECG), and the V6–V1 interpeak interval is similar to that in NS-LBBP.^2–4^ The term LB branch area pacing encompasses S-LBBP, NS-LBBP, and LVSP.

Deep septal pacing (DSP) is used to describe intraseptal pacing without the development of an RBBB-like pattern, as noted already. The ECG criteria of rapid LV activation are not present. LB fascicular pacing is sometimes used to describe direct pacing of the fascicles of the LB, rather than the common bundle itself. As the three fascicles form an arborizing network of conducting tissue, capture of fascicular tissue results in the engagement of the left conduction system in its entirety with antegrade impulse propagation, resulting in the rapid activation of the LV. The V6-RWPT is actually somewhat shorter than that achieved with proximal LB pacing as a portion of the LB–LV muscle conduction time, usually about 30 ms, is eliminated due to more distal conduction system capture.

A comparison of all these pacing modalities and their proper place in patient management is the focus of the intense ongoing research efforts by many groups. Almost all studies are observational and subject to selection bias, but trends are beginning to emerge, which will need to be confirmed by controlled studies.

Some facts are fairly clear. Biventricular pacing cardiac resynchronization therapy (CRT) procedures require more time and fluoroscopic exposure. The success rate is high (>95%), and the clinical response rate of about 70% compares very favorably with medical therapy. The CSP methods vary in complexity but are generally shorter and require less fluoroscopy. The pacing thresholds are remarkably low compared to CRT, and the clinical response rate equals CRT, while the echocardiographic response rate (73%) was found to be superior,^3^ albeit in a population less sick than the usual CRT controlled trial populations. Pujol-López et al. did compare CRT and CSP in a small randomized trial of heart failure (HF) patients with LBBB and found no difference in echocardiographic response rates or several measures of interventricular and intra-LV mechanical dyssynchrony between the two groups.^5^

The Multicentre European Left Bundle Branch Area Pacing Outcomes Study (MELOS)^6^ provides a snapshot of current CSP techniques. This was an observational registry of CSP conducted at 14 European centers, enrolling 2553 patients.

The implant success rate was a respectable 92.4% (82.2% in the more challenging HF patients). Fascicular LB pacing was performed in 65%, LVSP was performed in 26.5%, and traditional proximal septal S-LBBP/NS-LBBP was performed in only 9% of patients. This reflects the ongoing trend to place the LB pacing lead more distally, beyond the tricuspid valve leaflets, where the target is larger and tricuspid damage is less likely.

Is LVSP an acceptable form of CRT pacing? The case for LVSP is based on a 1978 tissue bath study of conduction velocity in canine tissue, which showed that conduction in the LV endocardium was more rapid than in the deeper layers of the myocardium, though not as rapid as in the specialized conduction tissue.^7^ Using ultra-high-frequency ECG and vectorcardiography, Curila et al. and Heckman et al. showed that LVSP produces slightly worse electrical synchrony and less interventricular dyssynchrony than LBBP.^8,9^ Mills et al. also showed that the hemodynamic effects of LVSP were superior to those of RV pacing and close to normal in the canine model.^10^ Pacing the left septal endocardium certainly eliminates transseptal conduction delay and it is undeniably simpler to perform as well. DSP is even simpler; the lead only needs to be screwed deep into the septum, and there is no requirement to demonstrate even ECG evidence (qR or RsR in V1), let alone rapid depolarization of the LV. The efficacy of this method remains to be determined.

Two groups compared LVSP, LBBP, and CRT in well-done observational studies, with patients being followed long term,^11,12^ and their findings regarding LVSP were similar. Diaz et al. found that there was a 31.4% absolute increase in freedom from HF-related hospitalizations (83% vs. 51.6%) with LBBP compared to LVSP, but no difference in mortality was found.^11^ Zhu and colleagues found that both HF hospitalizations and mortality were significantly increased in LVSP compared to LBBP (78% difference in the composite endpoint of mortality and HF hospitalizations).^12^

What can we learn from this? Compared to RV pacing, LVSP causes less dyssynchrony, and it may be an acceptable form of pacing in patients with normal or near-normal hearts; however, in patients with significantly impaired ventricular function, it may not compare well with LBBP or CRT. Both CRT and LBBP, selective or non-selective, correct the underlying pathology in LBBB, which is delayed activation of the posterolateral LV wall. Whether LVSP can do the same remains to be seen. Controlled trials will be needed to delineate its role in the future.

These two trials also compared LBBP to CRT and found statistically significantly fewer hospitalizations with LBBP but no difference in mortality. We must analyze this comparison carefully and with a degree of caution. The evidence for the efficacy of CRT in the HF population with wide QRS is based on numerous randomized controlled trials conducted on thousands of patients over decades. The American College of Cardiology (ACC)/American Heart Association (AHA)/Heart Rhythm Society (HRS)/European Heart Rhythm Association (EHRA) guidelines consider it a class 1 recommendation in the appropriate patient. It is understandably difficult for a treating physician to avoid choosing CRT for the sickest patients, leading to selection bias in an observational study. We must await randomized controlled trials comparing these two pacing modalities. Undoubtedly, each has its place. LBBP is less efficacious in patients with septal scar and disease of the conduction system distal to the stimulation site. CRT is limited by coronary vein anatomy, and its efficacy is reduced in the presence of lateral wall scar.^13^ Computer modeling by Strocchi et al. echoed these findings and also suggested that slowed intramyocardial conduction, by disease or drugs, seemed to favor LBBP over CRT.^14^ In addition, epicardial pacing reverses the normal endocardial-to-epicardial activation pattern. The disadvantages of each therapy are minimized by combining them, but at the cost of increased procedure complexity and fluoroscopic exposure.^15^

Two groups reported on the strength–duration curve of the LB. Hedgwood presented early data characterizing the strength–duration curve of the LB in a small number of patients.^16^ The author found that the chronaxie of the LB was significantly shorter than the RV endocardium (0.25 vs. 0.77 ms). Their results were later confirmed by Kiełbasa et al.^17^ in a larger group of patients, who measured the LB chronaxie to be 0.37 ms, revealing it to be significantly shorter than the RV endocardium. These findings support the concept that LB pacemakers should be programmed at shorter pulse widths than conventionally used for RV and LV epicardial pacing to minimize battery drain.
